# β, β‐Dimethylacrylshikonin Triggers Caspase‐Mediated Cell Apoptosis via Heme Oxygenase‐1 Upregulation and ERK/p38 Activation in Prostate Cancer

**DOI:** 10.1111/jcmm.71241

**Published:** 2026-06-08

**Authors:** Wei‐Chun Weng, Yi‐Hsien Hsieh, Chih‐Hsin Tang, Shian‐Shiang Wang, Chun‐Chuan Su, Shih‐Chi Su, Shun‐Fa Yang

**Affiliations:** ^1^ Division of Urology, Department of Surgery Tungs' Taichung Metroharbor Hospital Taichung Taiwan; ^2^ Department of Nursing Jenteh Junior College of Medicine, Nursing and Management Miaoli Taiwan; ^3^ Department of Post‐Baccalaureate Medicine, College of Medicine National Chung Hsing University Taichung Taiwan; ^4^ Institute of Medicine Chung Shan Medical University Taichung Taiwan; ^5^ Department of Medical Research Chung Shan Medical University Hospital Taichung Taiwan; ^6^ Department of Pharmacology, School of Medicine China Medical University Taichung Taiwan; ^7^ Department of Medical Laboratory Science and Biotechnology Asia University Taichung Taiwan; ^8^ Chinese Medicine Research Centre China Medical University Taichung Taiwan; ^9^ Division of Urology, Department of Surgery Taichung Veterans General Hospital Taichung Taiwan; ^10^ Whole‐Genome Research Core Laboratory of Human Diseases Chang Gung Memorial Hospital Keelung Taiwan; ^11^ Department of Medical Biotechnology and Laboratory Science, College of Medicine Chang Gung University Taoyuan Taiwan

**Keywords:** apoptosis, dimethylacrylshikonin, prostate cancer

## Abstract

β, β‐Dimethylacrylshikonin (DMAS), a natural naphthoquinone derivative isolated from the root of a biennial herb belonging to the family *Boraginaceae*, has been demonstrated to exhibit anti‐cancer and anti‐inflammatory features. Yet, the effects of DMAS on restraining the progression of prostate cancer (PC) remain mostly elusive. Here, we attempted to examine whether DMAS hampers PC progression and subsequently explored the underlying mechanisms. Our results demonstrate that DMAS elicited cytotoxicity to PC cell lines, DU‐145 and PC‐3 cells, along with promotion of apoptosis and cell cycle arrest. Moreover, fluctuations in the levels of many potential apoptosis markers (upregulation of HO‐1 and downregulation of cIAP‐1 and XIAP) and activation of caspase pathways were observed in DMAS‐treated PC cell lines. Furthermore, DMAS‐induced caspase activations in PC cells were affected by silencing of HO‐1 and the pretreatment with a selective ERK (U0126) or p38 inhibitor (SB203580), unveiling a functional linkage of HO‐1, ERK, and p38 signalling to the responses of DMAS‐treated PC cells. In conclusion, our results revealed that DMAS induced activation of caspase cascades to elicit cell apoptosis in PC, through HO‐1 upregulation and ERK/p38 activation. These findings provide possible avenues for the use of a naturally occurring compound with therapeutic values in fighting prostate carcinogenesis.

## 1 Introduction

Prostate cancer (PC) remains the second most prevalent neoplasm among males on a global scale and represents a great proportion of cancer‐related fatalities [1]. A high heterogeneity in the risks for PC has been identified, including age‐related, genetic, inflammatory and infectious, androgen‐related, dietary, and ethnic factors that contribute to PC susceptibility [2]. Of these established PC risks, familial and genetic factors are among the strongest, as current progress in DNA sequencing has revealed a plethora of PC predisposition genes that are functionally involved in DNA repair processes [3]. In addition to the determination of disease etiologies, multiple options for PC treatment have been implemented to improve the prognosis of local and metastatic PC [4]. Active surveillance, robotic‐assisted radical prostatectomy, stereotactic radiotherapy, and brachytherapy generally yield a favourable outcome for patients with localised diseases but may have adverse effects such as urinary symptoms and sexual dysfunction [5]. For recurrent and metastatic PC, salvage radiotherapy, chemotherapy, and androgen deprivation therapy are used to extend survival, since advanced PC frequently progresses despite androgen ablation and is considered incurable [6]. As such, the addition of androgen receptor pathway or poly(ADP‐ribose) polymerase inhibitors has demonstrated efficacy in patients with metastatic PC resistant to traditional hormonal therapy [7]. These prognostic observations underscore the unmet need to explore alternative treatment modalities that can address these challenges, thus prompting us to discover natural and effective compounds for PC management.

β, β‐Dimethylacrylshikonin (DMAS) was first isolated from the root of a biennial herb belonging to the family *Boraginaceae* (*Onosma paniculata* Bureau & Franchet) and shown to be most active in triggering cytotoxicity against melanoma cells among its other analogues [8]. It has been reported that DMAS displays a range of oncostatic features in various cancer types via numerous and interrelated cellular and molecular mechanisms. In breast cancer cells, DMAS has been found to inhibit proliferation through suppressing the activity of the NF‐κB pathway [9] and to potentiate the sensitivity of an extremely aggressive form to chemotherapeutic agents [10]. Not only being beneficial for the resistance to chemotherapy, but DMAS also showed a synergistic effect with an inhibitor directed against the epidermal growth factor receptor (EGFR) signalling, conferring cytotoxicity toward brain cancer cells [11]. In in vitro settings, the growth of lung cancer cells was inhibited by the treatment with DMAS via the activation of the p38 pathway [12]. Moreover, this phytochemical compound was also reported to induce apoptosis of a slowly growing bone cancer type, accompanied by activation of ERK and AKT [13]. Even though this in vitro and in vivo evidence has revealed an effect of this natural compound on counteracting cancer progression, knowledge regarding the influence of DMAS on PC still falls behind that on other types of neoplasms. In this study, we aimed to determine whether DMAS hinders PC progression and subsequently investigated the molecular mechanisms underlying DMAS's actions. Our results support the application of a naturally occurring constituent with therapeutic potential in combating PC.

## 2 Materials and Methods

### 2.1 Cell Culture and Reagents

Human PC cell lines, DU‐145 and PC‐3, were purchased from the American Type Culture Collection (Manassas, VA, USA) and cultured in RPMI 1640 Medium (Thermo Fisher Scientific, Waltham, MA, USA) supplemented with 10% fetal bovine serum (FBS) (Thermo Fisher Scientific, Waltham, MA, USA), 1% penicillin/streptomycin (Sigma‐Aldrich, St. Louis, MO, USA) at 37°C in a humidified atmosphere of 5% CO2. β, β‐Dimethylacrylshikonin (DMAS), of HPLC grade with ≥ 98% purity, was purchased from Sigma‐Aldrich (St. Louis, MO, USA) and dissolved in DMSO (Sigma‐Aldrich). U0126 was obtained from Cell Signalling Technology (Danvers, MA, USA), and JNK‐IN‐8 and SB203580 were purchased from Calbiochem (San Diego, CA, USA).

### 2.2 Measurement of Cell Viability/Proliferation

Cell viability/proliferation was evaluated with a microculture tetrazolium (MTT) colourimetric assay (Sigma‐Aldrich) as previously described [14]. PC cell lines were incubated with DMAS at different concentrations in 24‐well plates for 24 h, and cell viability was measured based on the production of formazan following solubilisation with isopropanol, which was recorded spectrophotometrically at 563 nm in a spectrophotometer (DU640, Beckman Instruments, Fullerton, CA).

### 2.3 Flow Cytometry Analysis

Cell cycling and apoptotic response of DMAS‐treated PC cells were assessed according to levels of cellular DNA and flipping of annexin V, respectively, by using flow cytometry as described previously [15]. Briefly, DU‐145 or PC‐3 cells were treated with DMAS for 24 h, and cellular DNA was labelled with propidium iodide (PI) (Thermo Fisher Scientific, Waltham, MA, USA) and analysed with a BD AccuriTM C6 Plus personal flow cytometer (BD Biosciences, San Jose, CA, USA). For determining apoptotic response, levels of annexin V located on the cell surface were monitored by using an FITC‐labelled Annexin‐V/PI Apoptosis Detection kit (BD Biosciences, San Jose, CA, USA). Cells treated with DMAS for 24 h were collected, stained with FITC‐conjugated annexin V and PI for 20 min in the dark, and analysed via flow cytometry.

### 2.4 Profiling of Apoptosis‐Related Proteins

Levels of apoptosis‐related proteins in DMAS‐treated PC cells were investigated by using a Proteome Profiler Human Apoptosis Array Kit (R&D Systems, Minneapolis, MN, USA) [16]. Protein extracts of DMAS‐untreated and ‐treated DU‐145 and PC‐3 cells were harvested and subjected to the apoptosis‐related protein array following the manufacturer's instructions. Relative expression of apoptotic markers was quantified by measurement of pixel density and normalisation to the signal of reference array spots.

### 2.5 Immunoblotting

PC cells were treated with/without a kinase inhibitor (U0126, JNK‐IN‐8, or SB203580) for 2 h or an siRNA for 48 h and subsequently incubated in the presence or absence of DMAS for 24 h. Total protein lysates (20 μg) were harvested and subjected to SDS‐PAGE analyses [17]. HRP‐conjugated secondary antibodies were obtained from Dako Corporation, Carpinteria, CA, USA. Densitometry data of immunoblots were generated and analysed by ImageJ software.

### 2.6 Gene Silencing

For transient knockdown of HO‐1, specific siRNA (small interfering RNA) targeting HO‐1 was designed with the BLOCK‐iT RNAi Designer (Thermo Fisher Scientific) [18]. PC cells were transfected with specific siRNA directed against HO‐1 or with control siRNA using the Lipofectamine 2000 transfection reagent (Life Technologies). The efficiency of gene silencing was assessed by immunoblotting, and cells were then subjected to DMAS treatment and the following experiments 48 h after transfection.

### 2.7 Statistical Analysis

Descriptive statistics are presented as the mean ± standard deviation (SD) from at least three independent experiments. Prior to statistical analysis, data distribution normality was assessed to ensure the appropriateness of parametric testing. Statistical significance between two groups was determined using Student's *t*‐test. A *p*‐value < 0.05 was considered statistically significant.

## 3 Results

### 3.1 DMAS Elicits a Dose‐Dependent Cytotoxicity to PC and Induces Cell Apoptosis in PC

**FIGURE 1:**
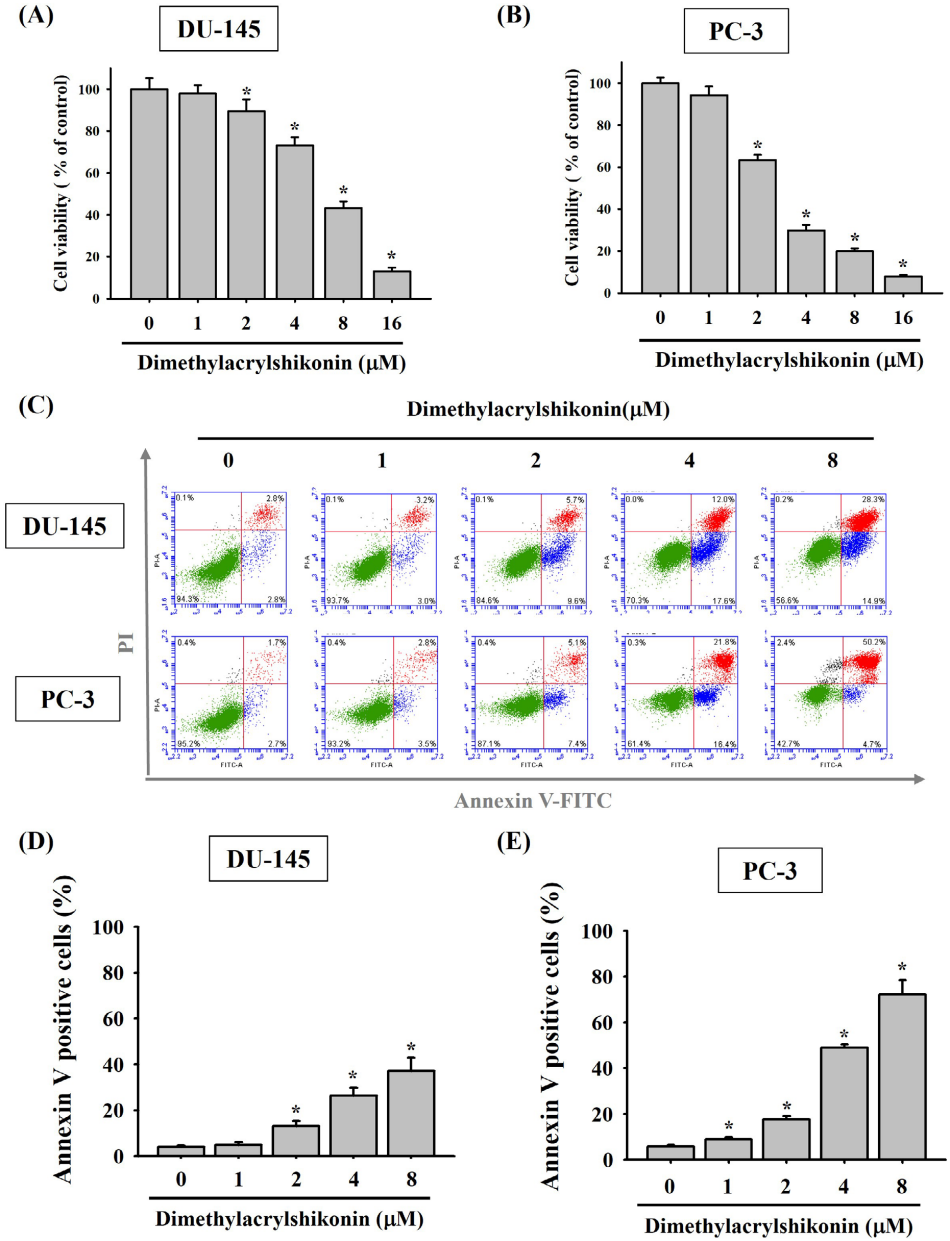
DMAS reduces the viability and triggers cell apoptosis in PC cells. The cytotoxicity of Dimethylacrylshikonin (DMAS) to PC cells. (A) DU‐145 and (B) PC‐3 cells were incubated with DMAS at various concentrations for 24 h and evaluated for cell proliferation. Results are demonstrated as the average ± SD from three separate experiments. *p < 0.05, compared with untreated controls using Student's t‐test. (C) DU‐145 and PC‐3 cells were incubated with DMAS at indicated concentrations for 24 h, stained with PI and annexin V, and analysed for apoptotic responses by flow cytometry. Data are representative of three independent experiments. (D, E) Quantification of apoptotic cell subsets in DMAS‐treated DU‐145 (D) and PC‐3 cells (E) as determined by the proportion of PI/annexin V double‐positive cells. Results are shown as the average ± SD from three separate experiments. *p < 0.05, compared with untreated controls using Student's t‐test.

To clarify the impact of DMAS on PC cells, the viability of two human PC cell lines, DU‐145 and PC‐3, exposed to different concentrations of DMAS (1 to 16 μM) was tested. We found that treatment with DMAS triggered a dose‐dependent effect on diminishing the viability of both PC cell lines (Figure 1A,B). This result is in concordance with its tumour‐suppressive characteristics observed in other types of malignancies [9, 10, 11, 12, 13], highlighting an oncostatic role of DMAS in the progression of PC. Considering the finding that DMAS effectively restrained PC proliferation, we next explored whether such a tumour‐suppressive effect of DMAS is attributed to altered cell cycle progression or apoptotic responses in PC. By labelling DMAS‐treated cells with PI and annexin V, we observed an increased proportion of apoptotic cells (PI/annexin V‐double positive) under the treatment with DMAS (Figure 1C–E). These results indicate that the anti‐proliferative effect of DMAS on PC is accompanied by apoptotic responses.

### 3.2 DMAS Reshapes Apoptosis‐Related Proteome in PC

**FIGURE 2:**
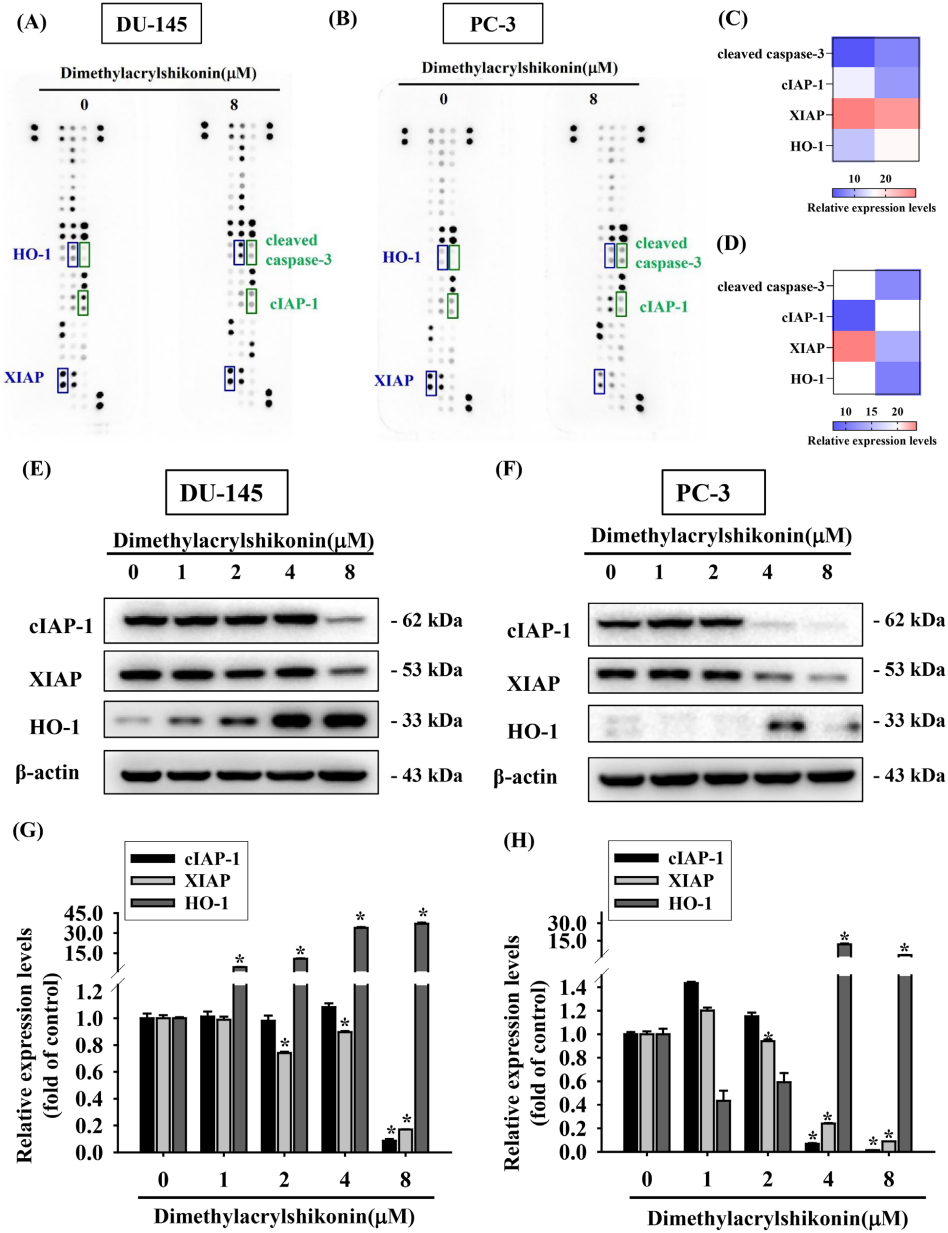
Protein regulatory landscape of apoptosis in DMAS‐treated PC cells. (A, B) Representative images of protein array membranes corresponding to DMAS‐untreated and ‐treated protein expression in DU‐145 (A) and PC‐3 cells (B). Spots of differentially expressed proteins are marked, labelled, and selected for further verification. (C, D) Densitometric analyses of selected spots from DU‐145 (C) and PC‐3 samples (D). (E, F) DU‐145 (E) and PC‐3 cells (F) were treated with DMAS at various concentrations for 24 h and examined for the expression of indicated apoptosis‐related proteins by immunoblotting. (G, H) Signal intensity of individual proteins from DU‐145 (G) and PC‐3 cells (H) in each condition was quantified and normalised with internal controls (β‐Actin). The values represent the mean ± SD of three independent experiments. *p < 0.05, compared with untreated controls using Student's t‐test.

**FIGURE 3:**
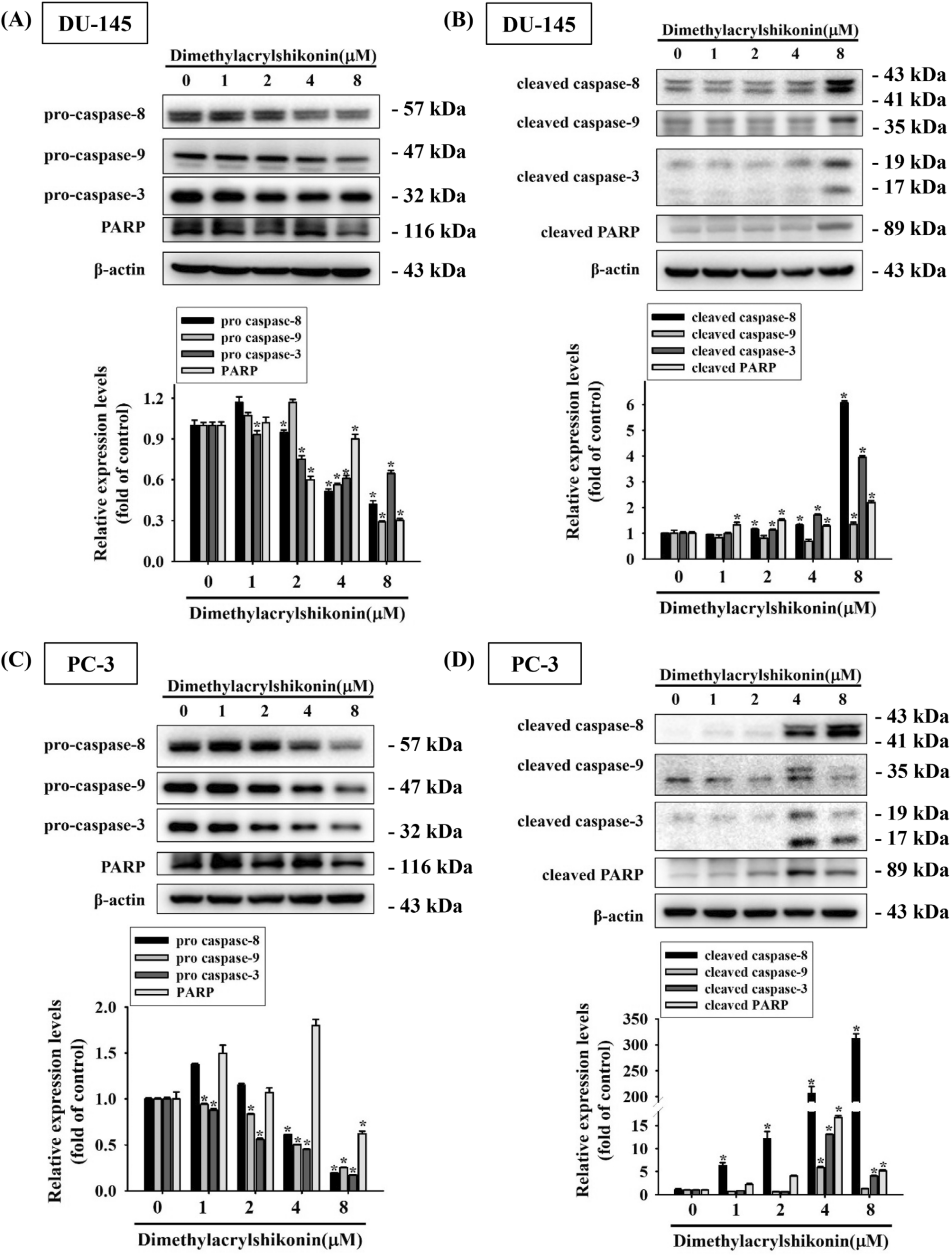
DMAS promotes the cleavage of pro‐caspase‐3, −8, and −9 in PC. DU‐145 (A, B) and PC‐3 cells (C, D) were treated with DMAS at the indicated concentrations for 24 h and assessed for the levels of intact (A, C) and cleaved forms (B, D) of caspases and PARP via immunoblotting. Comparisons of signal intensities were shown in the right panel. Data are the mean ± SD of three independent experiments. *p < 0.05, compared with untreated controls using Student's t‐test.

We further aimed to investigate the protein regulatory landscape of programmed cell death in DMAS‐treated PC cells by performing an array analysis of 35 apoptosis‐associated proteins. A compatible pattern of these marker expressions between DU‐145 and PC‐3 cells was observed (Figure 2A–D). Specifically, the expression of cellular inhibitor of apoptosis protein‐1 (cIAP‐1) and X‐linked inhibitor of apoptosis protein (XIAP) was reduced in DMAS‐treated DU‐145 and PC‐3 cells, whereas the level of heme oxygenase‐1 (HO‐1) in both PC cell lines was augmented by DMAS treatment. Downregulation of cIAP‐1 and XIAP, as well as upregulation of HO‐1 in DMAS‐treated cells, was further verified as an alteration in expression levels was particularly overt at higher concentrations (8 μM) (Figure 2E–H), indicating that DMAS treatment, especially at high concentrations, attunes the apoptotic proteome in prostate cancer. Moreover, treatment with DMAS diminished the levels of inactive precursors of caspase‐3, −8, −9, and poly (ADP‐ribose) polymerase‐1 (PARP), along with elevated levels of active (cleaved) forms of these apoptosis enzymes in two cell lines (Figure 3A–D). These data unveil a distinct protein regulatory landscape of apoptosis in DMAS‐treated PC cells, highlighted by fluctuations of several tentative apoptosis markers and activation of caspase cascades.

### 3.3 HO‐1 Mediates DMAS‐Induced Caspase Activation in PC

**FIGURE 4:**
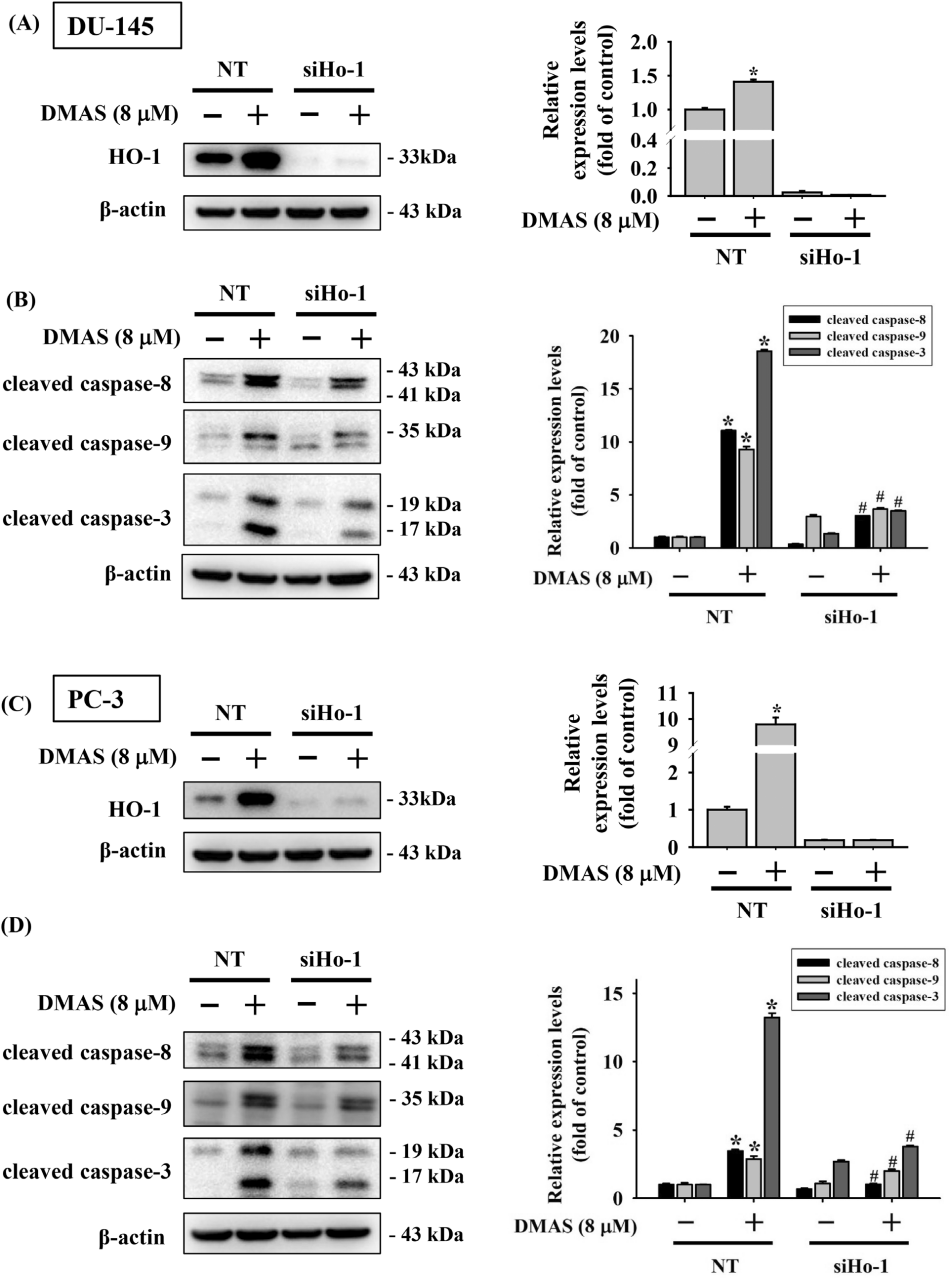
Silencing of HO‐1 reduces DMAS‐induced caspase cleavage in PC. DU‐145 and PC‐3 cells were treated with siRNAs against HO‐1 and a non‐target control for 48 h and subsequently incubated with/without DMAS for another 24 h. Protein lysates were prepared for evaluating the successful knockdown of HO‐1 in DU‐145 cells (A) and PC‐3 cells (C), and levels of cleaved forms of caspases via immunoblotting in DU‐145 cells (B) and PC‐3 cells (D). Densitometric analysis of immunoblots was conducted and shown in the right panel. Data represent the mean ± SD of three independent experiments. *p < 0.05, compared with untreated controls using Student's t‐test. #*p <* 0.05, compared with DMAS‐treated cells using Student's t‐test.

Since upregulation of HO‐1, a vital modulator of regulated cell death [19, 20, 21, 22], was noted in DMAS‐treated PC cells, we next tested whether HO‐1 is functionally involved in DMAS‐induced apoptotic responses of PC cells. To address this, silencing of HO‐1 expression was performed in DMAS‐treated and untreated PC cells. We demonstrated that specific knockdown of HO‐1 diminished DMAS‐induced cleavage of caspase‐3, −8, and −9 in DU‐145 and PC‐3 cell lines (Figure 4A–D). This finding suggests a functional connection of HO‐1 with DMAS‐induced cell apoptosis in PC.

### 3.4 ERK and p38 Signalling Contribute to DMAS‐Induced Caspase Activations in PC

**FIGURE 5:**
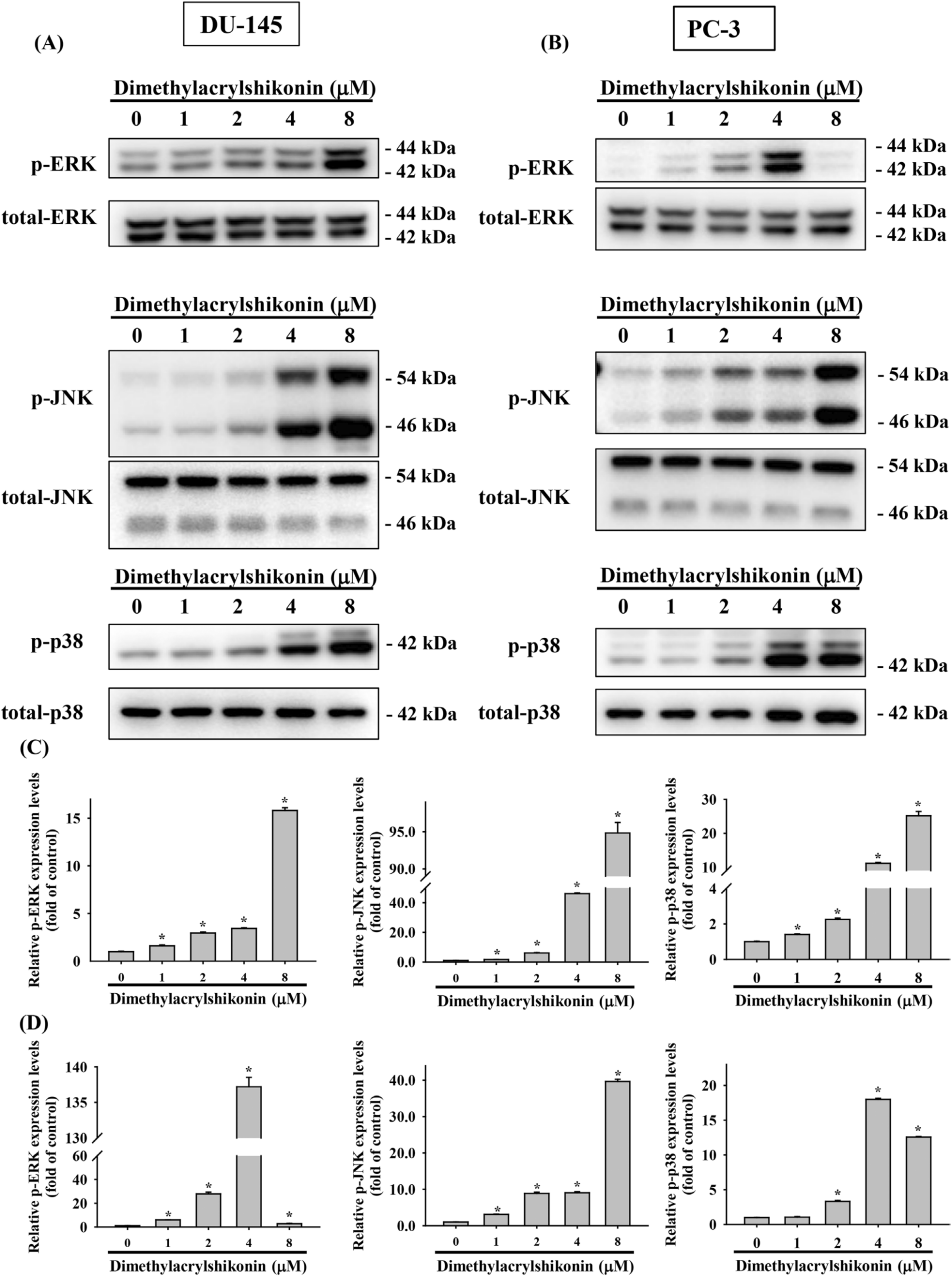
DMAS activates MAPKs in PC. DU‐145 (A) and PC‐3 cells (B) were treated with DMAS at indicated concentrations for 6 h and assessed for the phosphorylation status of ERK1/2 (ERK), JNK, and p38‐MAPK by immunoblotting. Quantification and comparison of signal intensities in each condition of DU‐145 (C) and PC‐3 cells (D). Data represent the mean ± SD of three independent experiments. *p < 0.05, compared with untreated controls using Student's t‐test.

**FIGURE 6:**
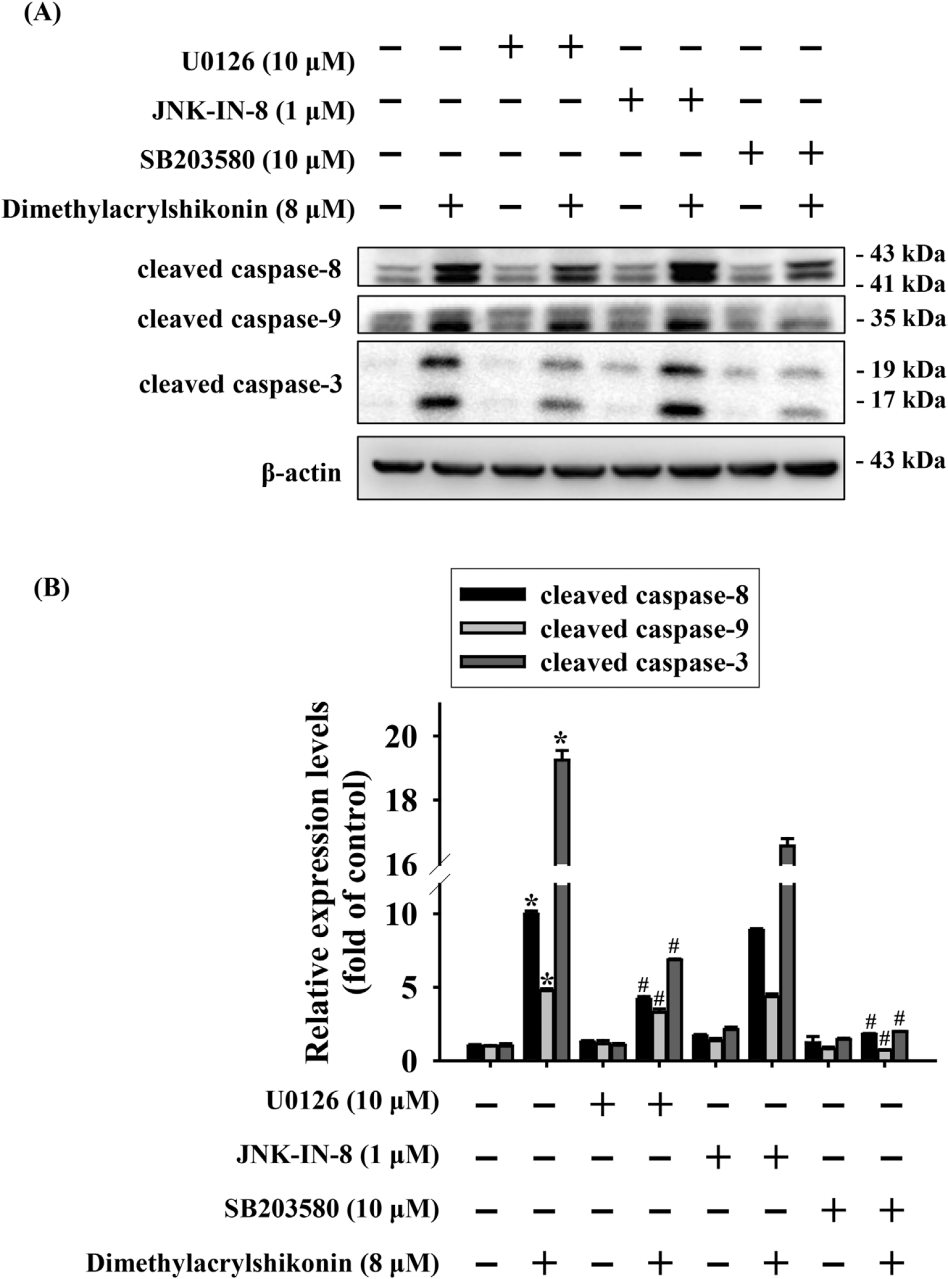
Functional involvement of ERK and p38 in DMAS‐induced caspase activation in PC. (A) DU‐145 cells were pretreated with the indicated kinase inhibitors for 2 h and then treated with DMAS for an additional 24 h. Cell lysates were prepared and evaluated for the levels of caspase cleavage by immunoblotting. (B) Densitometric analysis was performed, and signal intensities were normalised to internal controls (β‐Actin). Data are the average ± SD of three independent experiments. *p < 0.05, compared with untreated controls using Student's t‐test. #*p <* 0.05, compared with DMAS‐treated cells using Student's t‐test.

Mitogen‐activated protein kinases (MAPKs) are a family of serine/threonine protein kinases found to be crucial in directing apoptotic responses to a plethora of stimuli [23, 24, 25]. Therefore, we evaluated the levels of MAPK phosphorylation in DMAS‐treated PC cells and demonstrated that Erk1/2 (ERK), JNK, and p38 were significantly activated in both PC cell lines in response to DMAS (Figure 5A–D). This implicates DMAS as a potent external stimulus to transmit downstream signalling through MAPK activities in PC. To clarify the functional link of MAPKs to activation of caspase cascades in DMAS‐treated PC cells, we tested the influences of MAPK inhibition on DMAS‐induced caspase activations in PC cells. We found that pre‐treatment of DU‐145 cells with a selective ERK (U0126) or p38 inhibitor (SB203580) significantly reduced DMAS‐induced cleavage of pro‐caspase‐9, −8, and −3 (Figure 6A,B). However, interference with JNK activation by using JNK‐IN‐8 perturbed the status of caspase activations in DMAS‐treated DU‐145 cells (Figure 6A,B). These data indicate a functional involvement of ERK and p38 kinase in the activation of caspase cascades during the process of DMAS‐induced PC apoptosis.

## 4 Discussion

Contemporary treatment options of PC have yielded favourable outcomes for patients with localised cancer but may have adverse effects such as sexual dysfunction and urinary symptoms [5]. However, the prognosis of patients with advanced diseases remains a notable challenge. The implementation of complementary therapeutic strategies, thus, is needed to improve patients' survival and quality of life. It is mostly perceived that natural components isolated from medicinal herbs can elicit promising responses in cancer patients when given in combination with conventional medical care [26]. In this study, we demonstrated that DMAS, a natural naphthoquinone pigment isolated from the root of a biennial herb, induced apoptotic responses in PC cells. Further exploration of the molecular basis for DMAS's actions on PC cell death revealed a functional involvement of HO‐1 induction and caspase activation. Our results provide additional insights into the applications of this natural compound against prostate carcinogenesis.

DMAS has been reported for its therapeutic potential and has unique oncostatic features in comparison with its parent compound, shikonin [10], with DMAS generally exhibiting stronger anti‐cancer activities [27, 28]. Through various mechanisms, DMAS and its analogues inhibit the progression of numerous cancer types [28, 29]. In hepatocellular carcinoma and triple‐negative breast cancer, DMAS was demonstrated to induce the arrest of cell cycling at the G2 phase, thereby suppressing the tumour growth [10, 30]. In our investigation, we showed that DMAS promoted a substantial accumulation of PC cells at the sub‐G1 phase, which was consistent with the observation in DMAS‐treated melanoma cells [8]. During the progression of cell cycling, the sub‐G1 phase, characterised by massive DNA fragmentation and reduction in cellular DNA content, is strongly indicative of apoptosis [31]. Of note, induction of apoptotic responses, monitored by flipping of annexin V and activation of caspase cascades, was seen in DMAS‐treated PC cells. Similarly, treatment with DMAS triggered DNA fragmentation to induce cell apoptosis in liver cancer cells [30], while additional characteristics of cell apoptosis, including cell shrinkage, nuclear pyknosis and chromatin condensation, were also detected in DMAS‐treated breast cancer cells [9]. However, the inhibitory effects of DMAS on cancer progression are not limited to the induction of cell apoptosis but also involve other forms of programmed cell death. One example is that treatment with DMAS could circumvent cancer drug resistance by induction of necroptosis [28], a form of programmed necrosis that is mainly mediated by inflammatory regulators in a caspase‐independent manner [32]. In addition, DMAS was shown to trigger autophagy, a self‐degradative process through which cells break down and recycle unnecessary proteins and organelles to maintain intracellular homeostasis, in human lung adenocarcinoma cells [33]. Excessive or dysregulated autophagy can cause autophagic cell death, another caspase‐independent form of regulated cell death characterised by the accumulation of autophagosomes and functionally implicated in cancer development and treatment [34]. These findings, together with our results, highlight a tumour‐suppressive potential of DMAS against malignant diseases via multiple and interrelated cell death pathways.

Of note, in addition to activation of caspase cascades, upregulation of HO‐1 was observed in DMAS‐treated PC cells. Even though the effects of HO‐1 induction on suppressing cell proliferation and promoting apoptotic responses were documented [35, 36, 37], HO‐1 was commonly overexpressed in tumours as compared with adjacent non‐malignant tissues and found to mediate a cytoprotective response during cancer progression [38]. These contradictory findings suggest that HO‐1 might function in a tissue/stage‐specific manner and act as a promoter of PC cell apoptosis instead of a cytoprotective regulator in prostate carcinogenesis. Furthermore, treatment of PC cells with DMAS attuned MAPK signalling to confer the anti‐cancer responses. In a previous study, Wu et al. reported that DMAS suppressed the proliferation of triple‐negative breast cancer cells through inhibition of STAT3 phosphorylation [10]. In addition, pharmaceutical inhibition of p38‐MAPK blocked DMAS‐induced p38 activation and mitochondria‐dependent apoptosis in human lung adenocarcinoma cells [12]. Our data support the use of DMAS as a complementary treatment option against prostate cancer, employing a unique signature of apoptotic proteome via the ERK/p38 activation.

Our study demonstrated that DMAS exerts anti‐cancer effects in PC cells by promoting apoptosis. However, several limitations should be noted. First, although DMAS showed anti‐cancer activity in vitro [10, 27, 30], its pharmacological effects after absorption and metabolism in vivo remain unclear. Future animal studies are needed to further validate the tumour‐suppressive role of DMAS in PC. Second, both cell lines used in this study are AR‐negative or express minimal non‐functional AR. Since androgen receptor signalling plays a central role in PC progression, additional studies using AR‐positive PC models are warranted to further clarify the effects of DMAS and improve the generalisability of our findings.

**FIGURE 7:**
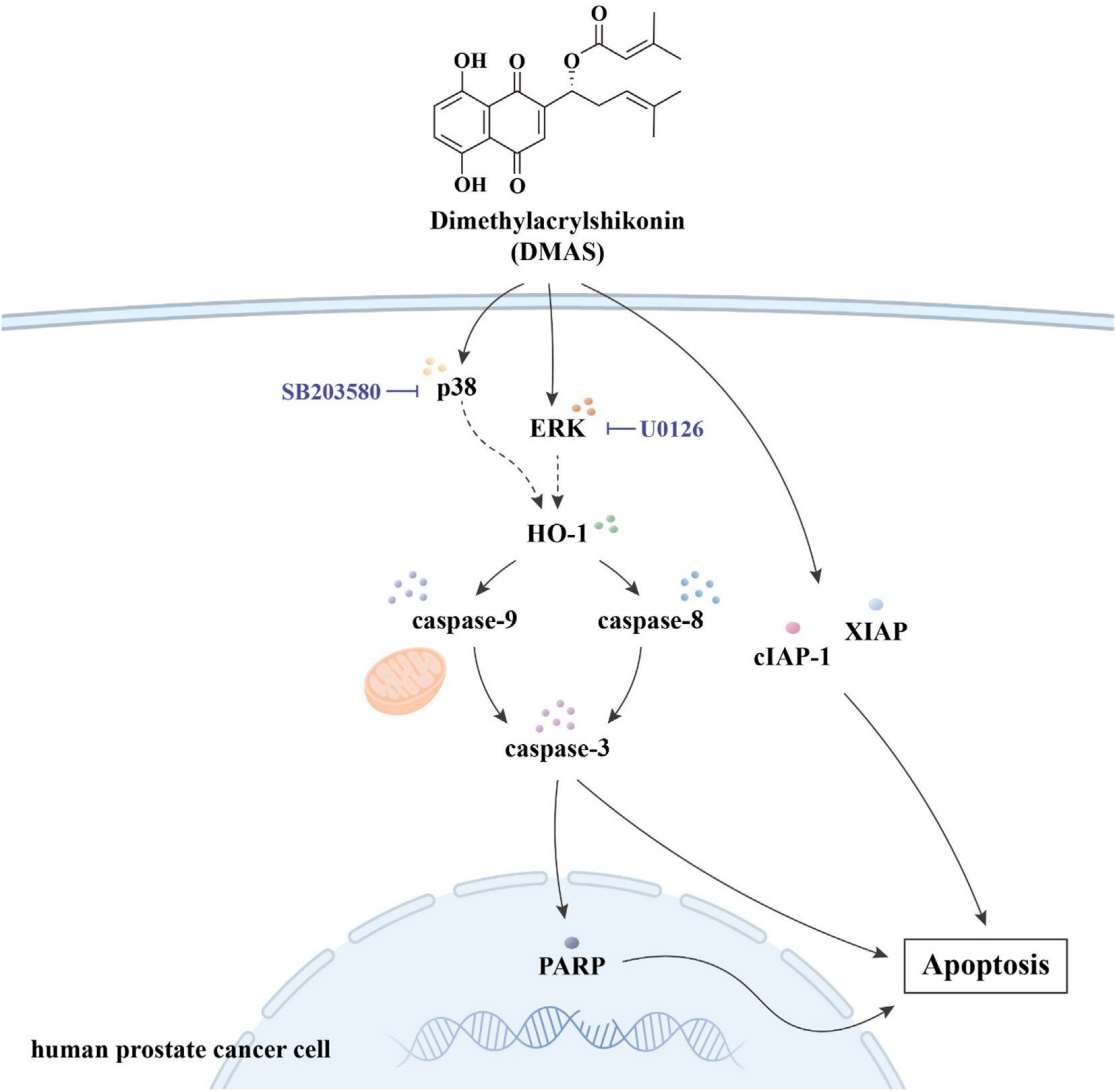
Schematic diagram of DMAS in the regulation of PC apoptosis.

In conclusion, we showed that DMAS activated caspase‐mediated programmed cell death in PC, through HO‐1 upregulation and ERK/p38 activation (Figure 7). Our findings provide novel insights into the therapeutic implications of a naturally occurring naphthoquinone, DMAS, in the management of prostate carcinogenesis.

## Author Contributions

**Wei‐Chun Weng:** conceptualization, data curation, writing – original draft, writing – review and editing. **Chih‐Hsin Tang:** data curation, investigation. **Shih‐Chi Su:** conceptualization, data curation, writing – original draft, writing – review and editing. **Shian‐Shiang Wang:** data curation, resources. **Shun‐Fa Yang:** conceptualization, data curation, writing – original draft, writing – review and editing. **Yi‐Hsien Hsieh:** conceptualization, investigation. **Chun‐Chuan Su:** data curation, investigation.

## Conflicts of Interest

The authors declare no conflicts of interest.

## Data Availability

The data that support the findings of this study are available from the corresponding author upon reasonable request.
